# Sociobiome - Individual and neighborhood socioeconomic status influence the gut microbiome in a multi-ethnic population in the US

**DOI:** 10.1038/s41522-024-00491-y

**Published:** 2024-03-11

**Authors:** Soyoung Kwak, Mykhaylo Usyk, Dia Beggs, Heesun Choi, Dariush Ahdoot, Feng Wu, Lorraine Maceda, Huilin Li, Eun-Ok Im, Hae-Ra Han, Eunjung Lee, Anna H. Wu, Richard B. Hayes, Jiyoung Ahn

**Affiliations:** 1 Perlmutter Cancer Center, NYU Langone Health, New York, NY USA; 2 Department of Population Health, NYU Grossman School of Medicine, New York, NY USA; 3 Nell Hodgson Woodruff School of Nursing, Emory University, Atlanta, GA USA; 4 Johns Hopkins University School of Nursing, Baltimore, MD USA; 5 Johns Hopkins University Bloomberg School of Public Health, Baltimore, MD USA; 6 Department of Population and Public Health Sciences, Keck School of Medicine, University of Southern California, Los Angeles, CA USA

**Keywords:** Microbiome, Policy and public health in microbiology

## Abstract

Lower socioeconomic status (SES) is related to increased incidence and mortality due to chronic diseases in adults. Association between SES variables and gut microbiome variation has been observed in adults at the population level, suggesting that biological mechanisms may underlie the SES associations; however, there is a need for larger studies that consider individual- and neighborhood-level measures of SES in racially diverse populations. In 825 participants from a multi-ethnic cohort, we investigated how SES shapes the gut microbiome. We determined the relationship of a range of individual- and neighborhood-level SES indicators with the gut microbiome. Individual education level and occupation were self-reported by questionnaire. Geocoding was applied to link participants’ addresses with neighborhood census tract socioeconomic indicators, including average income and social deprivation in the census tract. Gut microbiome was measured using 16SV4 region rRNA gene sequencing of stool samples. We compared α-diversity, β-diversity, and taxonomic and functional pathway abundance by SES. Lower SES was significantly associated with greater α-diversity and compositional differences among groups, as measured by β-diversity. Several taxa related to low SES were identified, especially an increasing abundance of *Prevotella copri* and *Catenibacterium sp000437715*, and decreasing abundance of *Dysosmobacter welbionis* in terms of their high log-fold change differences. In addition, nativity and race/ethnicity have emerged as ecosocial factors that also influence the gut microbiota. Together, these results showed that lower SES was strongly associated with compositional and taxonomic measures of the gut microbiome, and may contribute to shaping the gut microbiota.

## Introduction

Lower socioeconomic status (SES) is related to increased incidence and mortality due to chronic diseases, including cancer, cardiovascular disease, and diabetes^1–3^. While socioeconomic inequalities in health are well-established, the biological mechanisms that underlie SES-related health disparities are not well understood. Low SES is associated with multiple health-related behaviors, such as reduced access to medical and dental care^4^, increased engagement in unhealthy behaviors such as smoking and alcohol dependency^5^, and decreased engagement in positive health behaviors such as healthy eating and exercise^6^. We and others proposed that the gut microbiome may mediate the relationship between SES and chronic disease, because of growing evidence showing that the gut microbiome is often impacted by these same factors^7–9^.

The gut microbiome is largely established by the fourth year of life^10^ and there is abundant evidence that maternal and family SES influence the infant and childhood gut microbiome^11–17^. An important indicator that SES might also influence the gut microbiota in adulthood comes from studies showing that twin pairs who experience a different SES in adulthood also tend to exhibit a differential gut microbiome^18^; furthermore, multi-generational studies indicate that heritability plays only a minor role in gut composition of family members^19^. Recent studies of the gut microbiome in adults from the United States (U.S.)^20^, China^21^, and the United Kingdom (U.K.)^18^ also point to SES-related gut microbiome differentials at the population level; however, these studies were limited by small sample size (*n* = 44)^20^, limited microbial characterization^21^, or study population homogeneity^18^. Therefore, there is a need for larger studies that consider individual and area-related measures of SES in racially diverse populations^9,22^.

In a U.S. study of 825 participants of diverse race and nativity, the Food and Microbiome Longitudinal Investigation (FAMiLI) study, we investigated whether low SES, assessed by individual and neighborhood characteristics, is associated with overall gut microbiota diversity and composition and with specific microbial taxon abundances. We also explored whether nativity and race/ethnicity additionally influence the gut microbiome. As the FAMiLI study was specifically designed to include diverse populations by race and nativity, we had the unique opportunity to evaluate SES—microbiome relationships in the context of a widely diverse population.

## Results

### Study participants

The current analysis included 825 adults (36.7% male), with a mean age of 59.6 years (Table 1). The racial and ethnic group composition was 311 (37.7%) non-Hispanic White, 287 (34.8%) non-Hispanic Asian, 89 (10.8%) non-Hispanic Black, and 138 (16.7%) Hispanic participants. Of the participants, 48.1% were foreign-born and 25.0% had education to high school graduation or less. The majority of participants were never smokers (65.1%), followed by former smokers (24.5%) and current smokers (9.5%). Exercise was categorized as the average time spent per week: 13.6%, 23.8%, 30.5%, 31.0% for none, 1 hr/week, 2–3 h/week, and 4 hr/week, respectively. The mean body mass index (BMI) of the participants was 27.4 kg/m^2^, and the mean dietary acculturation index was -0.001, with a range from -0.315 (indicating lower acculturation) to 0.292 (indicating higher acculturation). The mean value was 0.062 for non-Hispanic White participants, -0.089 for non-Hispanic Asian participants, 0.026 for non-Hispanic Black participants, and 0.024 for Hispanic participants among the major racial/ethnic groups (Supplementary Table 1). The range and the number of participants in each quintile of occupational socioeconomic index (OSEI), neighborhood income, and social deprivation index (SDI) were presented. The two individual-level SES indicators (education and OSEI index) were strongly correlated with each other (Spearman ρ = 0.41, *p* value < 0.001), as were the two neighborhood-level indices (neighborhood income and SDI) (Spearman ρ = 0.81, *p* value < 0.001). Comparing individual-level to neighborhood-level measures showed correlations ranging from 0.27 to 0.39. Individual- and neighborhood-level SES tended to be correlated with nativity and race/ethnicity (Cramer’s V > 0.14, *p* value < 0.001). Also, nativity and race/ethnicity were very strongly correlated with each other (Cramer’s V = 0.86, *p* value < 0.001, Supplementary Fig. 2).

**Table 1:** Characteristics of Study Participants

Variable	Overall (*N* = 825)
**Age**	
Mean (SD) [min, max]	59.6 (11.1) [40, 91]
**Sex**	
Male	303 (36.7%)
Female	522 (63.3%)
**Nativity**	
U.S.-born	428 (51.9%)
Foreign-born	397 (48.1%)
**Race/Ethnicity**	
Non-Hispanic White	311 (37.7%)
Non-Hispanic Asian	287 (34.8%)
Non-Hispanic Black	89 (10.8%)
Hispanic	138 (16.7%)
**Smoking status**	
Never	537 (65.1%)
Former	202 (24.5%)
Current	78 (9.5%)
Missing	8 (1.0%)
**Exercise**	
None	112 (13.6%)
1 h/week	196 (23.8%)
2–3 h/week	252 (30.5%)
4 h/week	256 (31.0%)
Missing	9 (1.1%)
**Dietary acculturation index**	
Mean (SD) [min, max]	-0.001 (0.120) [-0.315, 0.292]
Missing	44 (5.3%)
**Body mass index, kg/m**^**2**^	
Mean (SD) [min, max]	27.4 (6.4) [15.5, 55.6]
Missing	20 (2.4%)
**Individual-level SES**	
**Education**	
More than high school graduate	614 (74.4%)
High school graduate or less; Low SES	206 (25.0%)
Missing	5 (0.6%)
**OSEI**	
Q5 [81.025, 92.782]	132 (16.0%)
Q4 [62.947, 80.919]	135 (16.4%)
Q3 [43.859, 62.573]	120 (14.5%)
Q2 [28.681, 42.994]	168 (20.4%)
Q1 [12.609, 28.645]; Lowest SES	139 (16.8%)
Missing	131 (15.9%)
**Neighborhood-level SES**	
**Income**	
Q5 [86302, 209063]	165 (20.0%)
Q4 [63446, 85551]	165 (20.0%)
Q3 [51806, 63036]	165 (20.0%)
Q2 [36250, 51773]	164 (19.9%)
Q1 [11809, 36236]; Lowest SES	166 (20.1%)
**SDI score**	
Q5 [1, 21]	165 (20.0%)
Q4 [22, 48]	170 (20.6%)
Q3 [49, 74]	160 (19.4%)
Q2 [75, 91]	185 (22.4%)
Q1 [92,100]; Lowest SES	145 (17.6%)

Values are presented as the mean (SD) for continuous variables and as the number of counts and percentages for categorical variables, the range is in the brackets. Income variable was derived using median household income (dollars) in the past 12 months (B19013_001) in the census tract obtained from American Census Survey 2011–2015. OSEI Occupational Socioeconomic Index, SDI Social Deprivation Index

### Socioeconomic status and gut microbiome overall diversity

**Fig. 1:**
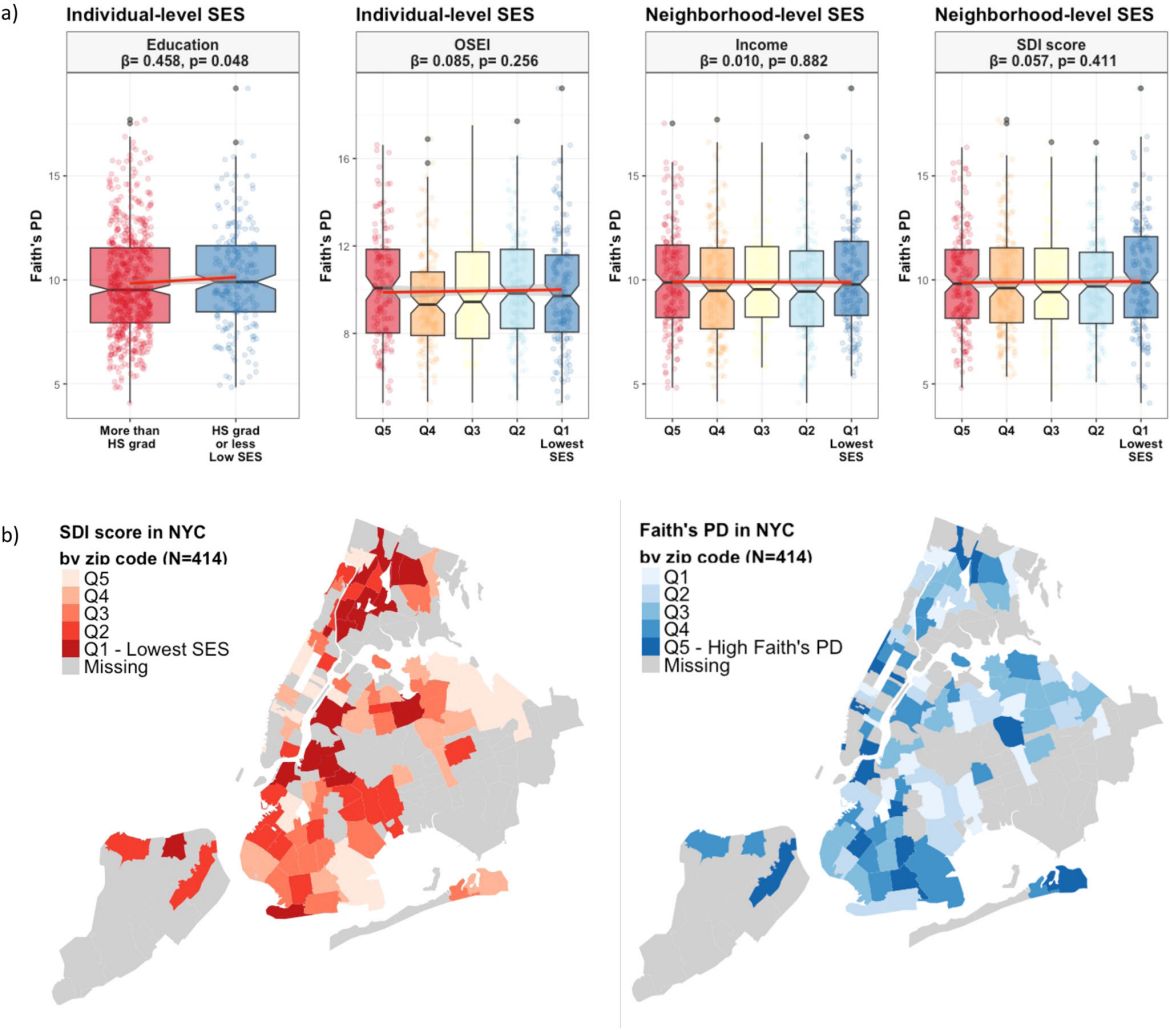
Alpha diversity by socioeconomic characteristics. **a** Faith’s phylogenetic diversity of 16S rRNA gut microbiome samples. Measures were compared using a null hypothesis of no difference between groups (Regression, *p* < 0.05). The regression model was adjusted for age, sex, smoking status, exercise, dietary acculturation index, and body mass index. The boxplot displays the median (center line inside the box), interquartile range (IQR, bounds of the box), minimums and maximums within 1.5 times the IQR (whiskers), and outliers (points beyond the whiskers). **b** Visual comparison of SDI score and Faith’s PD in NYC by zip code. Significant positive spatial autocorrelation was observed for SDI score and Faith’s PD (Moran’s I = 0.120, Moran’s I = 0.024, both *p* < 0.005). PD Phylogenetic diversity, HS grad High School graduate, OSEI Occupational Socioeconomic Index, SDI Social Deprivation Index.

Lower individual educational attainment was associated with greater microbial α-diversity represented as the number of phylogenetic tree-units within a sample (Faith’s phylogenetic diversity, *p* value < 0.05; Fig. 1a). Individuals living in areas of lower neighborhood income and/or greater neighborhood deprivation (SDI score) did not exhibit significantly greater α-diversity (Faith’s PD, *p-*value > 0.05, Fig. 1a). Among New York City resident participants of our study cohort (*n* = 414, 50.2%), positive spatial autocorrelation was observed for SDI score and microbiome Faith’s PD (Moran’s I = 0.120 for SDI score, and Moran’s I = 0.024 for Faith’s PD, both *p* < 0.05), indicating that areas with similar SDI score and Faith’s PD α-diversity tend to be located near each other within the New York City area (Fig. 1b).

**Fig. 2:**
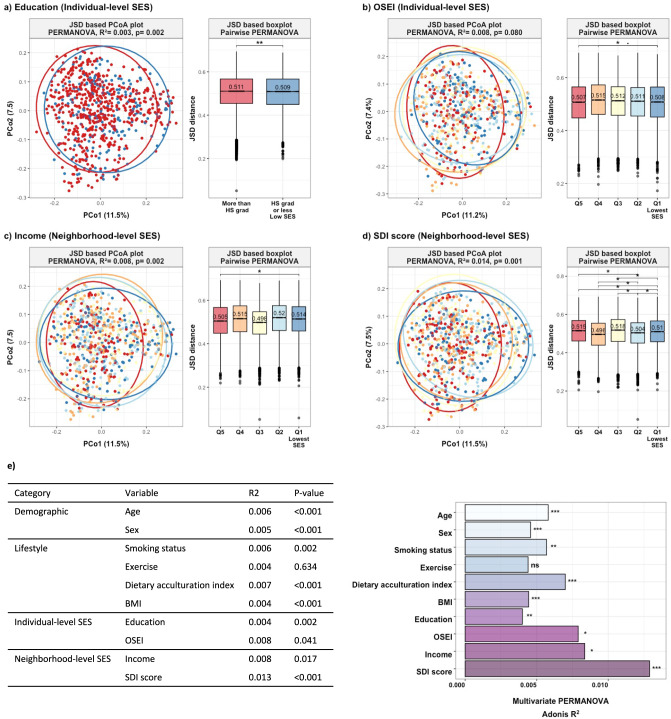
Beta diversity by socioeconomic characteristics. **a–d** Principal Coordinate Analysis (PCoA) plot and boxplot of the JSD distance. Statistical significance between socioeconomic indicators was determined using permutational multivariate analysis of variance (PERMANOVA) after adjusting for age, sex, smoking status, exercise, dietary acculturation index, and body mass index. The significance of differences among the groups was tested using pairwise-PERMANOVA. **e** Multivariate PERMANOVA model. The bars depict the amount of variance (R^2^) explained by each socioeconomic variable in JSD distance. Size effect and statistical significance were calculated by PERMANOVA including sociodemographic variables in one model. Stars denote the level of significance (Bonferroni post-hoc-tests; •*p* value < 0.1; *p value < 0.05; **p value < 0.01; ***p value < 0.001). HS grad: High School graduate; OSEI: Occupational Socioeconomic Index, SDI: Social Deprivation Index.

Consistent with findings for α-diversity, overall composition differentials in gut microbiome (β-diversity) were identified with respect to individual- and neighborhood-level SES indicators, as shown in principal coordinate plots and age, sex, smoking status, exercise, dietary acculturation index, and BMI adjusted Jenen-Shannon Divergence (JSD) boxplots (Fig. 2a-d, PERMANOVA: *p* value < 0.05). In the multivariate permutational multivariate analysis of variance (PERMANOVA) model (Fig. 2e), including multiple correlated SES indicators (Supplementary Fig. 2), all four SES indicators remained significantly associated with microbiome β-diversity. Notably, SDI score had the largest explanatory power on gut microbiome composition (R^2^ = 0.013) than other SES indicators (R^2^ were 0.004 to 0.008).

### Socioeconomic status and differential gut microbiome taxa

**Fig. 3:**
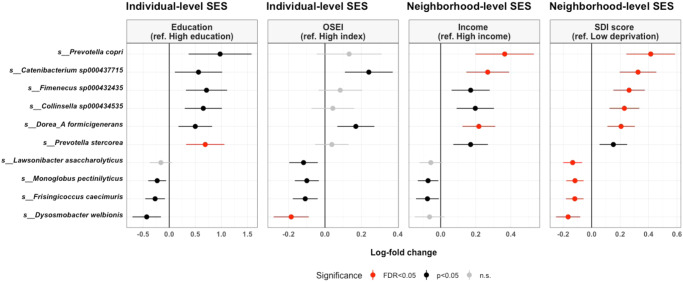
Differential abundance by socioeconomic characteristics. Forest plot showing the log-fold changes (x-axis) by species (y-axis) derived from the ANCOM-BC model, with 95% confidence interval error bars. ANCOM-BC was conducted by each socioeconomic indicator after adjusting for age, sex, smoking status, exercise, dietary acculturation index, and body mass index. Each dot is colored by the significance level. Log-fold change values greater than 0 indicate the fold change increase in the low SES (deprived) groups, while log-fold change values less than zero indicate the fold change decrease in the low SES groups.

Analysis of compositions of microbiomes with bias correction (ANCOM-BC) analysis further revealed several gut bacterial species associated with lower SES status (Fig. 3). Ten species were identified as differentially abundant by SES indicators, including 1 species by education, 1 by occupation, 3 by neighborhood income, and 9 by SDI score (False discovery rate, FDR < 0.05). SDI score identified the greater number of differential species, and this may be partially explained by the PERMANOVA results, that SDI score had the largest explanatory power on the gut microbiome composition. Lower SES-associated taxa include *Prevotella copri, Catenibacterium sp000437715, Fimenecus sp000432435, Collinsella sp000434535*, *Dorea_A formigenerans*, and *Prevotella stercorea* (FDR < 0.05) and higher SES-associated taxa include *Dysosmobacter welbionis, Frisingicoccus caecimuris*, *Monoglobus pectinilyticus*, and *Lawsonibacter asaccharolyticus* (FDR < 0.05). Notably, *Prevotella copri*, *Catenibacterium sp000437715*, and *Dorea_A formicigenerans* were associated with both neighborhood income and SDI score. *Dysosmobacter welbionis* was associated with both occupation and SDI score, with the largest log-fold change difference among higher SES-associated taxa.

**Fig. 4:**
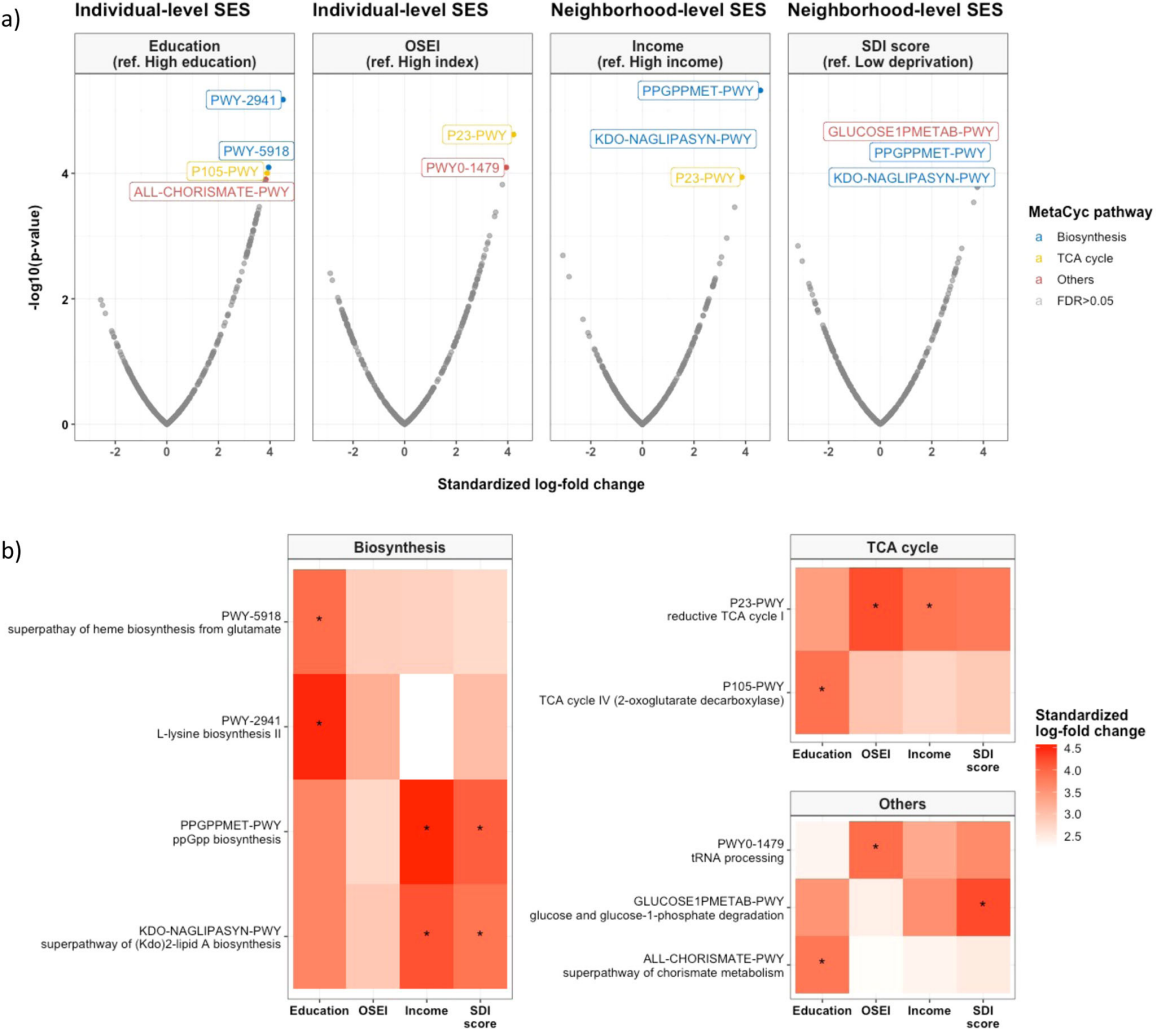
Deprivation of socioeconomic status and functional pathway. Functional pathways were predicted from 16S rRNA gene-based microbial compositions using the PICRUSt2 algorithm to make inferences from the MetaCyc pathway database. **a** Volcano plot showing the standardized log-fold changes (x-axis) by the negative log-transformed *p*-value (y-axis) derived from the ANCOM-BC model. ANCOM-BC was conducted by each socioeconomic indicator after adjusting for age, sex, smoking status, exercise, dietary acculturation index, and body mass index. **b** Only functional pathways relating to low socioeconomic status are included in the heatmap. Stars denote the significance of the ANCOM-BC (* FDR < 0.05). OSEI: Occupational Socioeconomic Index, SDI: Social Deprivation Index.

Figure 4 depicts microbiota functional differences across SES status based on imputed pathways using the PICRUSt2 algorithm (Fig. 4). From a total of 391 MetaCyc pathways tested, 9 pathways related to SES were identified by ANCOM-BC after adjusting for age, sex, smoking status, exercise, dietary acculturation index, and BMI (FDR < 0.05), including 4 pathways by education, 2 by occupation, 3 by neighborhood income, and 3 by SDI score (Fig. 4a). The positive standardized log-fold changes suggested that low SES is related to an increase in certain functional pathways, including the biosynthesis, and tricarboxylic acid (TCA) cycle. All SES indicators demonstrated a similar positive association with these pathways, indicating a consistent trend across SES indicators (Fig. 4b).

### Effect of race/ethnicity in the relationship between SES and gut microbiota

**Fig. 5:**
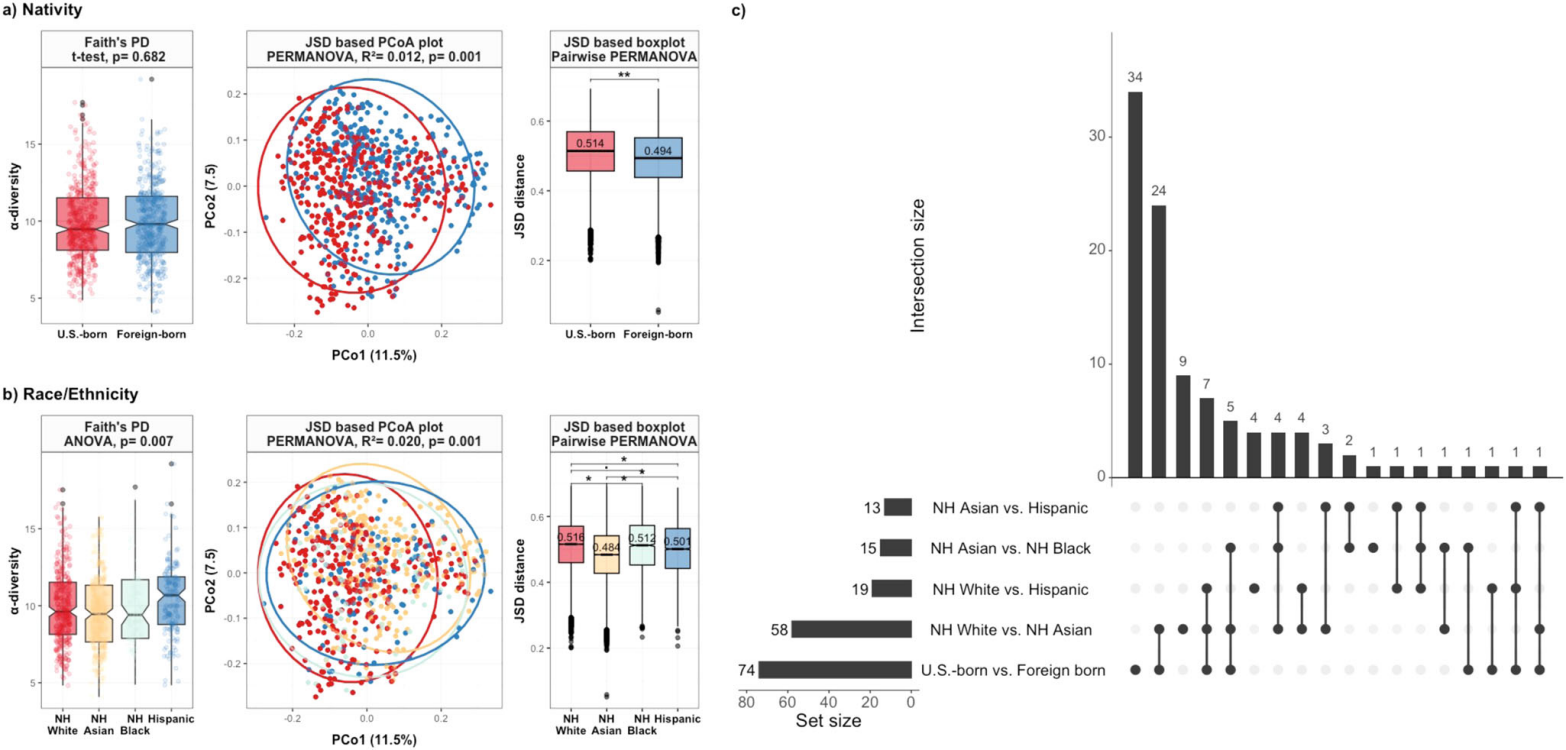
Microbiome profiles by nativity and race/ethnicity. **a** Nativity α-diversity and β-diversity **b** Race/ethnicity α-diversity and β-diversity **c** Upset plot showing the number of differentially abundant bacterial species identified via ANCOM-BC in individual comparisons of nativity and race/ethnicity, and shared species among various combinations of nativity and race/ethnicity. The set size on the left indicates the number of differential species in each comparison, while the connected dots indicate the common differential species across intersecting nativity and race/ethnicity comparisons. ANCOM-BC was conducted by nativity and race/ethnicity after adjusting for age, sex, smoking status, exercise, dietary acculturation index, and body mass index. NH: Non-Hispanic.

Black and Hispanic participants had lower SES (i.e., lower education, OSEI, neighborhood income and SDI score) (Supplementary Table 1). Foreign-born participants had significantly lower SES than U.S.-born participants. None of the SES indicators displayed significant heterogeneity by race/ethnicity (*p* value > 0.05). The Faith’s PD α-diversity did not differ between U.S.-born and foreign-born individuals, but overall β-diversity differed significantly between the nativity group (Fig. 5a). Between the racial/ethnic groups, both Faith’s PD and overall β-diversity significantly differed (Fig. 5b, all *p* < 0.05). With respect to nativity and race/ethnicity, a similar pattern in the differentially abundant species was observed when comparing non-Hispanic Asian participants to non-Hispanic White participants, and foreign-born individuals to U.S.-born individuals (Fig. 5c). Specifically, we observed 74 differentially abundant species when comparing U.S.-born to foreign-born individuals, and 58 species in the comparison between non-Hispanic White participants and non-Hispanic Asian participants. Among these comparisons, 24 species were found to be shared between the comparisons based on nativity and those between non-Hispanic White participants and non-Hispanic Asian participants.

## Discussion

Our study demonstrated that lower SES was positively associated with gut microbiome diversity and composition. Several bacterial taxa related to low SES were identified, especially among the *Prevotella copri*, *Catenobacterium sp000437715*, and *Dysosmobacter welbionis*. Nativity and race/ethnicity were additionally recognized as ecosocial factors^23,24^ encompassing various factors influencing the gut microbiota composition and diversity.

Our study demonstrated that lower SES is related to gut microbiome diversity and microbiome structure. Our findings that, all SES indicators were significantly associated with gut microbiome composition (β-diversity) is consistent with a large twin cohort in the United Kingdom^18^. Both studies suggest that gut microbiome β-diversity is moderately associated with both individual and neighborhood-level SES.

Directionality of SES and α-diversity association, however, remains inconsistent. Two previous studies in adults^18,20^ reported low SES is associated with reduced gut α-diversity^25^. Unlike our large multi-ethnic cohort, these studies were characterized either by the small sample size (*n* = 44)^20^, or homogenous population with low SES variability^18^. Our results are in line with other published literature linking SES and gut microbiota in children^11,14,16,17^ showing increased α-diversity related to low SES (i.e., comparing divergent socioeconomic schools, villages, area-based deprivation index, and maternal education). The similarity between childhood and adult microbiota is supported by the fact that the microbiota diversity, composition, and maturity tend to stabilize in the fourth years of life^10^, remaining so throughout life with further moderate modification by other environmental factors. Low SES has been associated in some settings with poor hygiene and a lack of sanitation which may lead to higher exposure to microorganisms and parasites and to increased α-diversity^26^. Our finding that Faith’s phylogenetic diversity showed association with SES may suggest that low SES is associated with a more unique, highly distinct microbial composition than is found in higher SES groups. More research is needed, however, to clarify and understand how SES relates to α-diversity.

We observed similar associations between several taxa and SES, as found in previous studies of adults^18,21^. Twin UK reported increased abundance of genus *Catenibacterium*, in the low neighborhood-level SES groups, similar to our findings^18^. In a study from China, the abundance of *Prevotella copri*, *Prevotella stercorea*, *Dorea formicigenerans*, and *Collinsella aerofaciens* was negatively associated with annual income^21^. We additionally compared the abundance of *Bacteroides* and *Prevotella* at genus level, which were noted to be a predictor of body weight^27^, and a biomarker for diet and lifestyle^28^. We found that low SES indicators were associated with increased abundance of *Prevotella*, and decreased abundance of *Bacteroides*, in line with other studies^20,21^. These differences in the abundance of *Bacteroides* and *Prevotella* may be explained by different dietary habits that are enriched in animal products relative to carbohydrates. The higher abundance of *Prevotella* in low SES has been reported in other studies and explains that higher intake of vegetables and fiber has been associated with^11,17^. *Dysosmobacter welbionis*, a recently identified human commensal bacterium^29^, has not been reported its association with SES. However, prior studies have demonstrated its potential role in preventing diet-induced obesity, diabetes, and metabolic disorders in mice^29,30^. Our findings of decreased *Dysosmobacter welbionis* abundance among low SES participants may be further connected to health outcomes such as diabetes and metabolic disorders. Additional comprehensive research is needed to establish a link between SES, microbiome, and health outcomes.

SES-related taxa were also related to nativity and race/ethnicity. Our earlier study revealed significant differences in microbiome composition across nativity and race/ethnicity^31^, including finding of differentially abundant *Prevotella copri* and *Catenibacterium*. Higher abundance of *Prevotella copri* was related to non-Western origin and diet, which is characterized by more consumption of high-fiber and low-fat diets than the typical Western diet. The abundance of *Prevotella copri* was enriched in foreign-born individuals, and in Asian and Hispanic individuals more than White individuals. The abundance of *Catenibacterium* was related to foreign-born Hispanic participants. The present work shows that these enriched species are also associated with low SES, especially with the neighborhood SDI score, even after additional adjustment of race/ethnicity.

We recognized nativity and race/ethnicity as ecosocial perspectives that encompass various cultural, social, and environmental factors that can potentially influence the microbiota indirectly. Other than dietary acculturation^31^ in relation to the nativity, factors such as cultural practices, migration-related psychosocial stress^32^, and healthcare access^33^ could contribute to the differences observed in gut microbiota profiles. In addition, the structural differences in SES factors, have been shown to have significant implications for population health outcomes^34–36^. The similarity in bacterial species differentials between U.S.-born vs. foreign-born individuals and Non-Hispanic White vs. Non-Hispanic Asian individuals could be attributed to the similarities within these respective groups. In our study, the majority of Non-Hispanic White individuals were U.S.-born, while most Non-Hispanic Asian individuals were foreign-born. When comparing racial/ethnic groups, there was no taxa differentially abundant between Non-Hispanic White vs. Non-Hispanic Black or Hispanic vs. Non-Hispanic Black individuals. These findings may be influenced by several factors, including the small sample size within the Non-Hispanic Black group. Although we cannot point out the exact reasons for these disparities, further studies with larger sample size to allow more comprehensive analysis by race/ethnicity and sex could help to clarify the observed differences. These factors are interconnected and multifaceted, and their effects on the gut microbiota may vary depending on individual circumstances.

Recently, the term “sociobiome” has been coined to describe the microbiota composition occurring in residents of a neighborhood or geographic region as a result of similar socioeconomic exposures^8^; socioeconomic status, but also broader social context, are of interest. In respect to social equity and health disparity, the socially minoritized populations are more likely to be exposed to environmental conditions that negatively affect health; including limited access to the fresh produce, poor access to the health care services, and poor hygiene^37^. In addition, the built environment and its related environmental exposures related to individual socioeconomic status (income, occupation) may impact the gut microbiota composition, diversity and function^38,39^. Therefore, understanding the sociobiome is warranted, and future studies should consider SES and the broader social context in identifying microbial factors to impact health inequalities. Furthermore, incorporating advanced techniques such as metagenomics and metabolomics could provide insights into the functional aspects of the microbiota.

Our study investigates the relationship between SES and the gut microbiome in a large racially and ethnically diverse population. The study adds to the body of knowledge on the impact of individual- and neighborhood-level SES on the gut microbiome. Even though the study was relatively large, a limitation remains that the distribution of SES in each racial/ethnic group tended to be limited. Additionally, the population distribution of our study cohort does not mirror the general population of the U.S. According to the U.S. Census data from 2016, the percentage of White, Asian, Black, and Hispanic individuals in the general population is reported as 61.2%, 13.3%, 5.7%, and 17.8%, respectively. In contrast, our study cohort consisted of 37.7%, 34.8%, 10.8%, and 16.7% for White, Asian, Black, and Hispanic individuals, respectively. Therefore, we are cautious in generalizing our findings to the broader U.S. population. Despite controlling for several key lifestyle variables in our hypothesis testing, there may still be unmeasured confounding factors that could contributed to the findings. Another limitation is that our interpretation of the findings is constrained by the available data.

In conclusion, our study demonstrated the significant association between SES and gut bacterial profiles across a diverse population. Differentials in SES were associated with α-diversity, β-diversity, the abundance of bacterial species, and microbial functions. Our results support the important role of SES in shaping gut microbiome composition.

## Methods

### Study population

Detailed information on the FAMiLI study population is available elsewhere^31^. Briefly, FAMiLI is an ongoing multi-ethnic prospective study in the U.S. that began in 2016. Participants aged 40 years or older were recruited, completed demographic and dietary questionnaires, and provided stool samples. This study was approved by the NYU Langone Health Institutional Review Board (#s12-00855), and all participants provided written informed consent. For the current analysis, we used previously sequenced stool samples from 873 participants recruited between 2016 and 2018 with available SES and demographic data and who did not use antibiotics in the 2 weeks prior to the stool collection^31^. Participants with missing or unknown data on age, gender, race/ethnicity or nativity (*n* = 10) were excluded from this analysis. We further excluded subjects with failed sequencing (*n* = 9), and insufficient stool sample gut microbial richness (*n* = 29), resulting in the final sample size of 825 subjects from varying racial/ethnic (White, Asian, Black, and Hispanic), and nativity (U.S.-born and foreign-born) backgrounds (Table 1). Specifically, race/ethnicity and nativity information were self-reported. Participants were asked to select their racial identification from categories including White, Black, Asian, Pacific Islander, American Indian or Alaskan Native, or not-identified/others. Additionally, participants were asked whether they identified as Hispanic origin or not. Based on their responses, we categorized the participants into the racial/ethnic groups of Non-Hispanic White, Non-Hispanic Black, Non-Hispanic Asian, and Hispanic. Nativity was determined based on participants’ self-reported information on their place of birth.

### Lifestyle variables

The smoking status of participants was categorized into three groups (never, former, and current). This categorization was based on their responses to two questions: “Have you ever smoked cigarettes regularly for six months or longer?” and “Do you smoke cigarettes regularly now?”. Participants who have never smoked were classified as “never smokers”. Those who smoked in the past but not currently were classified as “former smokers”, and those who currently smoke regularly were classified as “current smokers”. Exercise habit was classified into four groups based on their reported weekly exercise hours: none, 1 hour per week, 2–3 hours per week, and 4 or more hours per week. Dietary acculturation index derived from a published study on U.S. nativity and dietary acculturation^31,40^ was used to represent participants’ dietary information. Briefly, participants completed 137-item food frequency questionnaire. This index accounts for the participants’ nativity status and variations in dietary intake among different racial/ethnic groups. The index was calculated using the generalized UniFrac distance based on daily food frequencies and the hierarchical food categorization and was used to characterize dietary dissimilarities between participants in the study. The index’s values range from -0.315 to 0.292, with higher values indicating having a more acculturated diet, closely resembling the typical dietary patterns of U.S.-born White participants in our study. Body mass index (BMI) was calculated as measured weight divided by measured height squared (kg/m^2^).

### Socioeconomic status

Socioeconomic status (SES) is an individual’s relative social and economic position in relation to others^41,42^. Individual level SES is often characterized by measures of highest education, usual occupation, and/or income^42^. SES can also be conceptualized and measured at the neighborhood-level, that is where a person lives. Neighborhood-level SES may serve as a proxy for individual-level SES^43^, but it may also represent a separate environmental SES indicator, which may influence health outcomes independently of individual SES^44,45^. Herein, we examined two individual-level (education and occupational socioeconomic index) and two neighborhood-level SES indicators (neighborhood income and social deprivation index) that represent SES across these two domains.

Individual education and occupation were self-reported by questionnaire. The education level was classified as either a) high school graduate or less or b) more than high school. Participant’s self-reported usual occupation was matched to a corresponding standard occupational classification and U.S. Census Bureau’s coding scheme, and then assigned the occupational socioeconomic index (OSEI), which reflects the education, income, and prestige associated with an individual’s occupation^46^. The OSEI score was not assigned to those who did not provide their occupation or were not classified in the Census Bureau coding scheme (i.e., homemakers, unemployed, others) (15.9%). The OSEI score ranges from 0–100, with lower values indicating greater deprivation.

The area-level SES indicators were derived from the Census Bureau’s American Community Survey (ACS) 5-year summary file (https://www.census.gov/programs-surveys/acs/). Participants’ addresses were geocoded using ArgGIS software (ESRI Inc, Redlands, CA) and coordinates were converted into census tract identifiers. The neighborhood income level was derived using median household income in the census tract (B19013_001: median household income in the past 12 months) obtained from ACS data for 2011–2015. The neighborhood social deprivation index (SDI), a well-validated index of SES^47^, is a composite measure of seven neighborhood SES characteristics from ACS data for the years 2011–2015: poverty, education, non-employment, living in a renter-occupied home, living in crowded housing, single-parent household, no car ownership. The SDI ranges from 0–100, with lower values indicating lower deprivation. For the analysis, SDI score was reversed to be ordered by low to high level of SES. The OSEI, neighborhood income, and reversed SDI were categorized into quintiles and were ordered from greatest (Q5) to least (Q1) to estimate the effect of lower SES on gut microbiome profiles.

### Fecal sample collection and microbiome assessment

Using a well-tested protocol, we have used in previous studies^31,48^, participants were given the necessary supplies and instructions to collect fecal samples. The stool samples were collected either using saran wrap or stool collection paper, or by using toilet paper just before completing a bowel movement. Two marble-sized fecal samples, of approximately 8 grams, were collected using a spoon attached to the cap of the sample collection tube. The cap was closed tightly with the samples on the spoon, shaken well, and then placed in a preaddressed package, for mailing within 24 hours. Upon arrival at NYU, the fecal samples were registered and stored at -80 °C. Stool samples underwent 16S rRNA gene sequencing at the Environmental Sample Preparation and Sequencing Facility at Argonne National Laboratory^31,48^. DNA was extracted using the PowerSoil DNA isolation kit (MO BIO Laboratories; Carlsbad, CA), following the manufacturer’s protocol. DNA was amplified for the V4 region of the 16S rRNA gene using the 515 F/806 R primer pair, which included Illumina flow cell adapter sequences with sample-specific barcodes^49^. Sequencing reads were demultiplexed and paired-end reads were joined, followed by quality filtering using the QIIME2 pipeline^50^. Next, the DADA2 workflow was applied, which used sequence error profiles to obtain high-quality amplicon sequence variants (ASVs)^51^. The ASVs were then assigned taxonomy using the Greengenes 2 reference database (released October 2022) through q2-greengenes plugin within QIIME2^52^. A phylogenetic tree was constructed by inserting the sequences into the Greengenes reference tree using the q2-fragment-insertion plugin within QIIME2^53^.

### Statistical analysis

Gut microbiome profiles characterized by α-diversity, β-diversity, and differential abundance of species were analyzed with respect to individual-level (education and occupational socioeconomic index) and neighborhood-level SES indicators (neighborhood income and social deprivation index), as well as nativity, and race/ethnicity. To calculate α- and β-diversity measures, data was rarefied to even sampling depth at a sequencing depth of 1000, where the rarefaction curve reached a sufficient plateau (Supplementary Fig. 1). We measured α-diversity as Faith’s phylogenetic diversity (PD, representing phylogenetic richness considering the similarity between bacteria based on shared evolution^54^). Alpha-diversity measure was calculated using ‘phyloseq’ package^55^, and was compared by sociodemographic indicators using linear regression adjusting for age, sex, smoking status (never/former/current), exercise (none/1 hr per week/2–3 h per week/4 h per week), dietary acculturation index (continuous), and BMI (continuous) as confounding variables, t-test or one-way ANOVA as appropriate for the respective measures. We measured β-diversity using the JSD distance^56^. Beta-diversity measures were calculated using the ‘vegan’^57^ and ‘phyloseq’ packages, and were compared using principal coordinates analysis (PCoA) and PERMANOVA^58^. The box plot of the JSD distance and pairwise PERMANOVA were used to, respectively, represent and test the significance of differences between groups. Multivariate PERMANOVA, including age, sex, and individual-, neighborhood-level SES indicators, and lifestyle confounding variables (smoking status, exercise, dietary acculturation index, and BMI) were constructed to compare the explained variance of each of the variables. We used Spearman correlation for ordinal variables and Cramer’s V for nominal variables to examine the correlations between sociodemographic indicators^59^.

For differential abundance analysis with respect to sociodemographic indicators, analysis of compositions of microbiomes was carried out with bias correction (ANCOM-BC)^60^, adjusting for age, sex, smoking status, exercise, dietary acculturation index, and BMI. We used a minimum prevalence filter of 10% and a FDR threshold of 0.05 when identifying significantly differentially abundant species. Functional pathways were imputed from 16SV4 region rRNA gene-based microbial compositions using the PICRUSt2 algorithm^61^, with reference to the MetaCyc pathway catalog^62^. A total of 391 MetaCyc pathways were imputed. Functional pathways relating to the SES indicators were identified by ANCOM-BC, controlling for age, sex, smoking status, exercise, dietary acculturation index, and BMI. The effect size of the ANCOM-BC identified imputed pathways were visualized in volcano plots and heatmaps. All analyses were conducted using R (4.1.0).

### Reporting summary

Further information on research design is available in the Nature Research Reporting Summary linked to this article.

### Supplementary information

Reporting Summary

Supplementary Information

## Data Availability

The 16S rRNA sequencing data that support the findings of this study have been deposited in the Sequence Read Archive (PRJNA559143), along with demographic metadata, to be released upon publication. Additional data on the study participants are available from the corresponding author upon reasonable request.

## Code availability

The underlying code for this study is not publicly available but may be made available to qualified researchers on reasonable request from the corresponding author.
