# Supplementary material for: Sociobiome - Individual and neighborhood socioeconomic status influence the gut microbiome in a multi-ethnic population in the US
Source: NPJ Biofilms Microbiomes. 2024 Mar 11;10:19. doi: 10.1038/s41522-024-00491-y (PMC10928180; doi:10.1038/s41522-024-00491-y)
Supplement: Supplementary file 2 — Supplementary Information [file 41522_2024_491_MOESM2_ESM.pdf]

Supplementary Information

Supplementary Table 1. Characteristics of Study Participants by Race/Ethnicity

	NH White (N=311)	NH Asian (N=287)	NH Black (N=89)	Hispanic (N=138)
**Age**				
Mean (SD) [min, max]	57.6 (10.1) [40, 87]	64.9 (11.6) [40, 91]	54.2 (8.8) [40, 80]	56.3 (9.6) [40, 81]
**Sex**				
Male	138 (44.4%)	93 (32.4%)	29 (32.6%)	43 (31.2%)
Female	173 (55.6%)	194 (67.6%)	60 (67.4%)	95 (68.8%)
**Nativity**				
U.S.-born	297 (95.5%)	8 (2.8%)	85 (95.5%)	38 (27.5%)
Foreign-born	14 (4.5%)	279 (97.2%)	4 (4.5%)	100 (72.5%)
**Smoking status**				
Never	185 (59.5%)	218 (76.0%)	55 (61.8%)	79 (57.2%)
Former	101 (32.5%)	47 (16.4%)	22 (24.7%)	32 (23.2%)
Current	25 (8.0%)	19 (6.6%)	12 (13.5%)	22 (15.9%)
Missing	0 (0%)	3 (1.0%)	0 (0%)	5 (3.6%)
**Exercise**				
None	30 (9.6%)	41 (14.3%)	7 (7.9%)	34 (24.6%)
1 hr/week	66 (21.2%)	61 (21.3%)	31 (34.8%)	38 (27.5%)
2–3 hr/week	110 (35.4%)	82 (28.6%)	28 (31.5%)	32 (23.2%)
4 hr/week	103 (33.1%)	101 (35.2%)	22 (24.7%)	30 (21.7%)
Missing	2 (0.6%)	2 (0.7%)	1 (1.1%)	4 (2.9%)
**Dietary acculturation index**				
Mean (SD) [min, max]	0.062 (0.093) [-0.261, 0.292]	-0.089 (0.104) [-0.305, 0.288]	0.026 (0.095) [-0.253, 0.240]	0.024 (0.112) [-0.315, 0.238]
Missing	13 (4.2%)	14 (4.9%)	4 (4.5%)	13 (9.4%)
**Body mass index, kg/m^2**				
Mean (SD) [min, max]	28.4 (6.5) [17.9, 55.0]	23.9 (3.4) [15.5, 40.3]	31.3 (7.4) [15.8, 55.6]	29.7 (6.8) [16.8, 51.0]
Missing	0 (0.0%)	14 (4.9%)	4 (4.5%)	2 (1.4%)
**Individual-level SES**				
**Education**				
More than high school graduate	294 (94.5%)	186 (64.8%)	81 (91.0%)	53 (38.4%)
High school graduate or less; Low SES	17 (5.5%)	98 (34.1%)	8 (9.0%)	83 (60.1%)
Missing	0 (0.0%)	3 (1.0%)	0 (0.0%)	2 (1.4%)
**OSEI**				
Q5 [81.025, 92.782]	85 (27.3%)	30 (10.5%)	10 (11.2%)	7 (5.1%)
Q4 [62.947, 80.919]	89 (28.6%)	20 (7.0%)	18 (20.2%)	8 (5.8%)
Q3 [43.859, 62.573]	49 (15.8%)	40 (13.9%)	12 (13.5%)	19 (13.8%)
Q2 [28.681, 42.994]	54 (17.4%)	69 (24.0%)	25 (28.1%)	20 (14.5%)
Q1 [12.609, 28.645]; Lowest SES	18 (5.8%)	63 (22.0%)	15 (16.9%)	43 (31.2%)
Missing	16 (5.1%)	65 (22.6%)	9 (10.1%)	41 (29.7%)
**Neighborhood-level SES**				
**Income**				
Q5 [86302, 209063]	93 (29.9%)	53 (18.5%)	11 (12.4%)	8 (5.8%)
Q4 [63446, 85551]	76 (24.4%)	71 (24.7%)	11 (12.4%)	7 (5.1%)
Q3 [51806, 63036]	66 (21.2%)	63 (22.0%)	17 (19.1%)	19 (13.8%)
Q2 [36250, 51773]	48 (15.4%)	50 (17.4%)	21 (23.6%)	45 (32.6%)
Q1 [11809, 36236]; Lowest SES	28 (9.0%)	50 (17.4%)	29 (32.6%)	59 (42.8%)
**SDI score**				
Q5 [1, 21]	126 (40.5%)	26 (9.1%)	8 (9.0%)	5 (3.6%)
Q4 [22, 48]	93 (29.9%)	62 (21.6%)	13 (14.6%)	2 (1.4%)
Q3 [49, 74]	53 (17.0%)	69 (24.0%)	25 (28.1%)	13 (9.4%)
Q2 [75, 91]	25 (8.0%)	112 (39.0%)	15 (16.9%)	33 (23.9%)
Q1 [92,100]; Lowest SES	14 (4.5%)	18 (6.3%)	28 (31.5%)	85 (61.6%)

Values are presented as the mean (SD) for continuous variables and as the number of counts and percentages for categorical variables, the range is in the brackets. Income variable was derived using median household income (dollars) in the past 12 months (B19013_001) in the census tract obtained from American Census Survey 2011–2015. NH: Non-Hispanic, OSEI: Occupational Socioeconomic Index, SDI: Social Deprivation Index

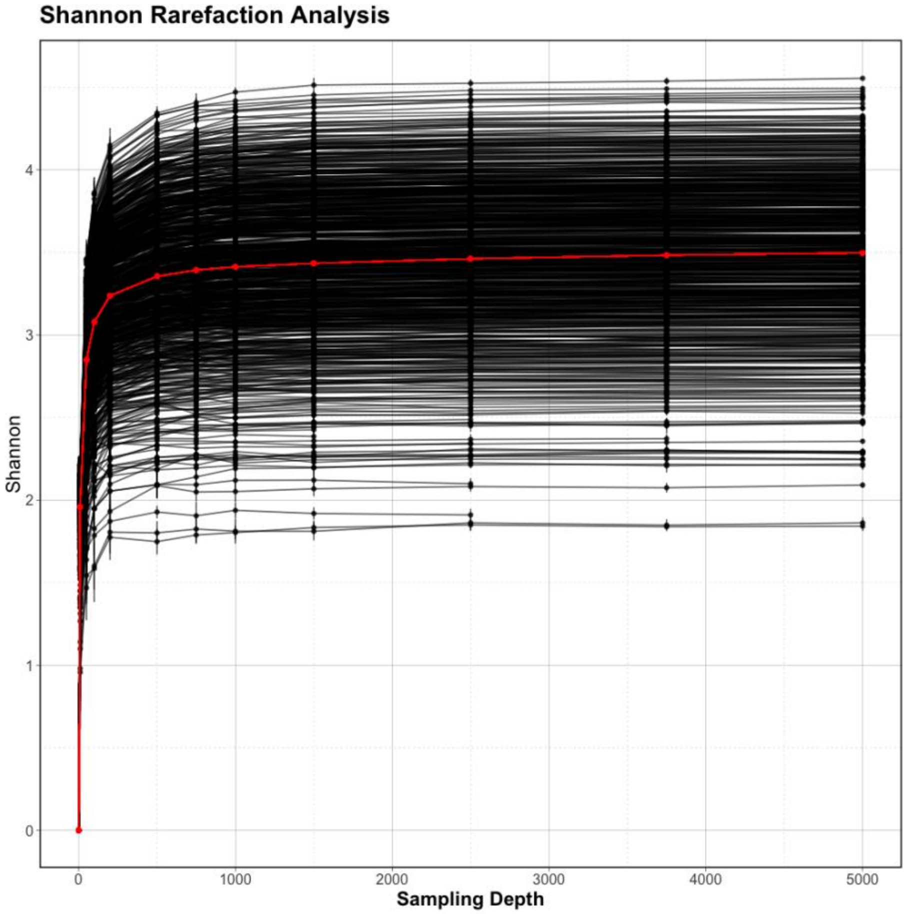

Supplementary Figure 1. Rarefaction curves of the Shannon index
The Shannon diversity index were calculated in 100 iterations at different rarefied sequencing depths (from 1 to 5000 sequence reads per sample), and averaged for each subject at each sequencing depth. Each subject is represented by a black line, while the red line represents the avarage values for all subjects. At the rarefaction depth of 1000 sequences per sample, 23 out of 825 samples (2.8%) were excluded for diversity analysis.

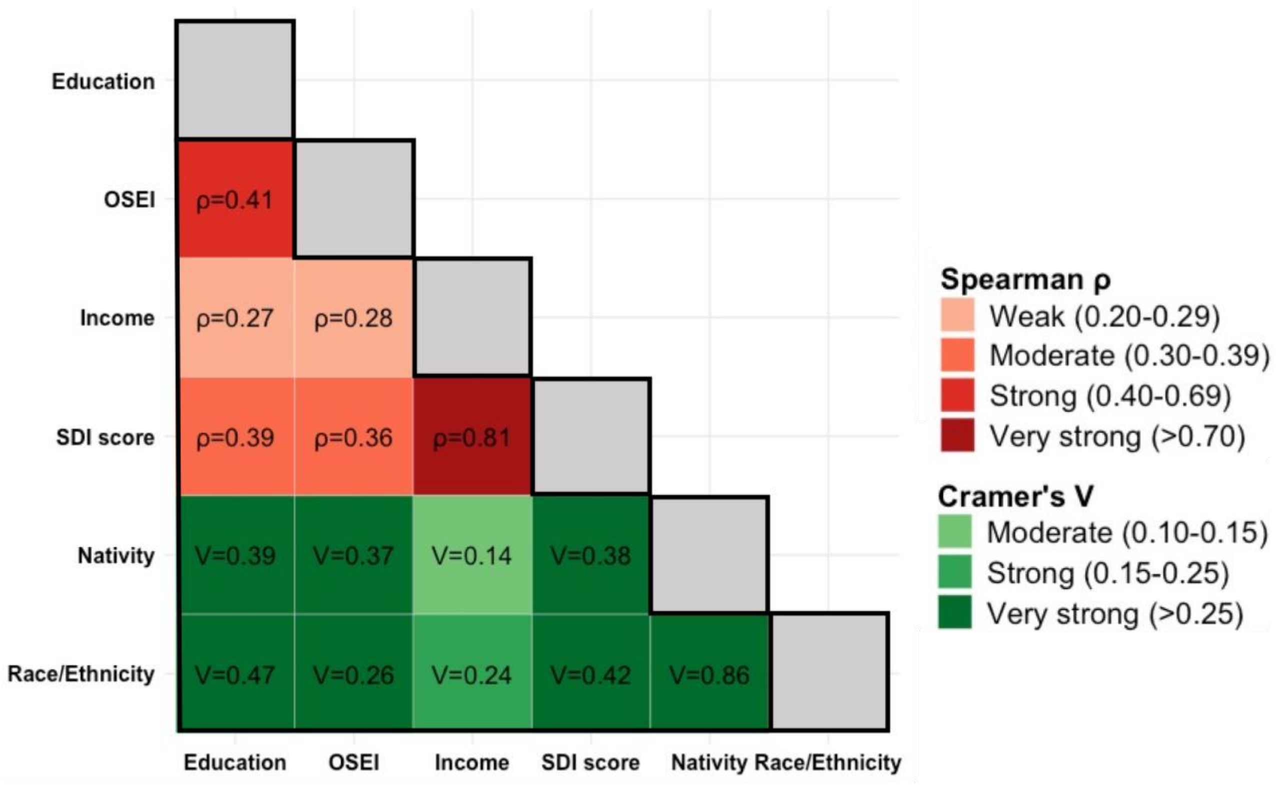

Supplementary Figure 2. Correlation between Socioeconomic Status, Nativity, and Race/Ethnicity
The heatmap provides a visualization of the correlation structure among the variables, employing Spearman corrleation for ordinal variables and Cramer's V for nominal variables, respectively. The color intensity reflects the magnitutde of the correlation effect size. Data are from the full sample (N=825) of the FAMiLI study. OSEI: Occupational Socioeconomic Index, SDI: Social Deprivation Index
